# Severe fatigue and related factors in cancer patients before the initiation of treatment

**DOI:** 10.1038/sj.bjc.6604739

**Published:** 2008-10-21

**Authors:** M M Goedendorp, M F M Gielissen, C A H Verhagen, M E J W Peters, G Bleijenberg

**Affiliations:** 1 Expert Centre Chronic Fatigue, Radboud University Nijmegen Medical Centre, 4628, P.O. Box 9101, 6500 HB, Nijmegen, The Netherlands; 2 Department of Medical Oncology, Radboud University Nijmegen Medical Centre, 452, P.O. Box 9101, 6500 HB, Nijmegen, The Netherlands

**Keywords:** depressive mood, diagnosis, fatigue, neoplasm, physical activity, sleeping problems

## Abstract

It is generally known that fatigue is a common symptom during cancer treatment, and in cancer survivors. However, fatigue was never studied after diagnosis, before cancer treatment was initiated. This study investigated the prevalence of severe fatigue, and related factors, in cancer patients before the initiation of treatment. One hundred and seventy-nine patients with various malignancies were assessed before start of treatment with curative intention, including the Checklist Individual Strength, Sickness Impact Profile, Beck Depression Inventory Primary Care, Symptom Checklist-90, and six Numeric Rating Scales to measure fatigue, pain and physical activity. To test which factors contributed to severe fatigue a logistic regression analysis was performed. In total 23.5% patients were severely fatigued, varying between diagnoses; prostate cancer (14.3%), breast cancer (20.3%), and gastrointestinal cancer (28.1%). Currently lower physical activity (*P*=0.013), more depressive mood (*P*=0.014), impaired sleep and rest during the day and night (*P*=0.045), and fatigue 1 year before diagnosis (*P*=0.005) contributed to severe fatigue. Relatively large numbers of cancer patients already experience severe fatigue before initiation of treatment, varying between 14–28%. The factors that contributed to severe fatigue at this stage were physical activity, depressive mood, impaired sleep and rest, and fatigue 1 year before diagnosis.

Fatigue is a frequently reported symptom in cancer patients and when severe, it is a distressing symptom interfering with daily functioning. Cancer patients experience fatigue at different stages during their illness. The prevalence of fatigue during cancer treatment ranges from 25 to 99% in different samples (Servaes *et al*, 2002a). After successful cancer treatment severe fatigue remains problematic in 19–38% of the disease-free cancer survivors (Servaes *et al*, 2002a; Prue *et al*, 2006).

It is generally thought that during the active period of cancer treatment, symptoms of fatigue arise as a consequence of the cancer itself, and the treatments patients receive, such as surgery, chemotherapy and radiotherapy. Other factors are also suggested to influence fatigue during cancer treatment. Psychological distress, such as depression, somatisation, anxiety, and also sleep quality were previously found to relate with fatigue (Servaes *et al*, 2002a; Respini *et al*, 2003). It is suggested that the experience of receiving cancer treatment in itself contributes to the development of fatigue (Hickok *et al*, 2005).

Fatigue in cancer patients prior to treatment has seldom been investigated. Some studies investigated fatigue before start of chemotherapy (Jacobsen *et al*, 1999; Ancoli-Israel *et al*, 2006; Berger *et al*, 2007) or radiotherapy (Smets *et al*, 1998; Stone *et al*, 2001; Hickok *et al*, 2005), but looking at these studies more closely revealed that most patients were not treatment naive. The majority of patients already received treatment that could have contributed to fatigue, such as surgery, hormone therapy, or chemotherapy.

Results of three quality of life studies indicate that fatigue might be problematic in treatment naive cancer patients (Cimprich, 1999; Handy *et al*, 2002; Visser *et al*, 2006). The first study found that lung cancer patients before surgery reported significantly more fatigue compared with age-matched control subjects (Handy *et al*, 2002). Two other studies concluded that fatigue contributed to increased distress and impaired quality of life in newly diagnosed cancer patients (Cimprich, 1999; Visser *et al*, 2006).

Cancer patients report that the period of diagnosis was very distressing, and research does confirm this. At diagnosis emotional functioning, anxiety, and sleep problems were the most problematic in patients with oral and oropharyngeal cancer (Shepherd and Fisher, 2004). In newly diagnosed breast cancer patients, disturbances in mood states and insomnia were also found, in addition to loss of concentration (Cimprich, 1999). Thus, newly diagnosed cancer patients have been studied in the past, but research specifically aimed at fatigue in this group is lacking.

The first objective of this study is to determine how many cancer patients report severe fatigue after being diagnosed, but before initiation of any medical treatment for cancer. If patients do report severe fatigue, the second objective is to establish which factors contribute to severe fatigue before cancer treatment, and whether mood, such as anxiety and depression, and sleep problems contribute to fatigue.

## Materials and methods

### Patients and procedure

Patients were recruited from one university hospital and six regional hospitals in the period from November 2005 until August 2007. Patients were included in this study after being diagnosed with a primary tumour and before initiation of treatment with curative intention. Treatment could be surgery, radiotherapy, chemotherapy, or a combination of these. Patients could additionally receive hormone therapy. In concordance with national and regional guidelines of the comprehensive cancer centre, the curability of a patient with cancer was determined, and the treatment procedure was chosen. All treatment options were discussed in the multi disciplinary working party for the specific tumour group, before the treatment procedure was decided. Patients in this study were recruited as part of a larger ongoing intervention study for fatigue during cancer treatment, and preventing chronic fatigue after finishing cancer treatment. To minimise drop out and exclusion during the ongoing study, patients with lung cancer, and head and neck cancer were not included. Patients were included if they were between 18 and 75 years old, and able to speak, read and write Dutch. Patients were excluded when having a co-morbidity that could cause fatigue, or when patients indicated to be severely fatigued for several years or have been seeking treatment for their fatigue. In addition, patients who were receiving psychiatric or psychological treatment in the last 3 months were excluded.

Eligible patients were informed about the study by their physicians and were asked if a researcher could approach them. When a specialised cancer unit was present in a hospital, such as a mamma care or colon care unit, specialised nurses checked for eligibility and informed patients. When patients agreed the physician informed the researcher. Patients who agreed to be approached received written information on the study and were contacted by telephone by the researcher or a test-assistant. When patients agreed to participate an appointment was made for the baseline assessment. The baseline assessment took place, at the Expert Centre Chronic Fatigue of the Radboud University Nijmegen Medical Centre, at the hospital where patients would receive treatment, or at the patients’ home. All participants gave their written informed consent before baseline assessment. The ethics committees from all seven involved hospitals gave approval for the study.

The data presented in this study are based on cancer patients who were treatment naive, and were assessed before initiation of treatment.

#### Instruments

Information on age, gender and diagnosis was provided by the patient's physician from patients who agreed to be approached, also from patients who did not participate eventually. From all participating cancer patients demographic and medical characteristics were gathered by self-report using questionnaires. Information on marital status and level of education were collected as part of the demographic data. The following information on medical characteristics was obtained: medication use in the past month, medical history on co-morbidities, and receiving psychological and psychiatric treatment during patients’ lifetime.

Fatigue severity was assessed by the subscale fatigue of the Checklist Individual Strength (CIS) (Vercoulen *et al*, 1994, 1999). The CIS is a well-validated instrument among patients with chronic fatigue syndrome (CFS) and in the working population (Beurskens *et al*, 2000; Dittner *et al*, 2004). The fatigue subscale consists of eight items scored on a seven-point Likert Scale, with scores ranging from eight to 56. Based on research with CFS patients a score of 35 or higher indicate severe fatigue (Vercoulen *et al*, 1994). A score between 27 (mean score for healthy adults plus one s.d.) and 35 indicate a heightened experience of fatigue (Vercoulen *et al*, 1999). The CIS was used in earlier research investigating cancer survivors (Servaes *et al*, 2001, 2002c, 2003; Gielissen *et al*, 2006).

Depression was assessed with the Beck Depression Inventory Primary Care (BDI-PC) (Beck *et al*, 1997b). This is a seven item questionnaire with scores ranging from zero to 21. A score of four or higher on the BDI-PC is indicative for a clinical depression (Beck *et al*, 1997a). The BDI-PC is based on a set of non-somatic items from the BDI-II (Beck *et al*, 1996).

Depressive mood was measured with the Symptom Checklist-90 (SCL-90) (Arrindell and Ettema, 1986), subscale depression. Sixteen items measure depressive mood, with scores ranging from 16 to 80. Higher scores indicated a stronger depressive mood.

Anxiety was measured with the SCL-90 subscale anxiety. Ten items measure anxiety with scores from 10 to 50. Higher scores indicated more anxiety.

Quality of nocturnal sleep was measured with the SCL-90 subscale sleep. Three items measure sleep with scores from three to 15. Higher scores indicated lower quality of sleep. In addition, the impact of the disease on sleep and rest during the night and day was measured with the subscale sleep/rest of the Sickness Impact Profile –8 (SIP) (Bergner *et al*, 1981; Jacobs *et al*, 1990). Higher scores on this subscale was an indication of more impairment on sleep/rest. Seven items measured impairments on sleep/rest, with scores ranging from zero to 499.

Physical activity was measured with an 11-point Numeric Rating Scale (NRS) ranging from zero to 10. Patients were asked how physically active they were in the period since diagnosis. Zero indicated ‘not physically active’ and 10 ‘physically very active’.

Pain was also measured with an 11-point NRS. Patients were asked how much pain they had experienced in the period since diagnosis, on a scale from zero to 10. Zero indicated ‘no pain’ and ten ‘very much pain’.

Patients were asked additionally to indicate their level of fatigue, physical activity, and pain before diagnosis retrospectively, 1 year before diagnosis and 3 years before diagnosis. Thus, in total six 11-point NRS’ were used, ranging from zero to 10.

### Statistical analysis

All data analysis was performed with SPSS (version 14.0). Differences between participating and non-participating cancer patients were tested with *χ*^2^. For the first objective descriptive statistics were used to describe demographic characteristics of treatment naive cancer patients, and the data on the presence of severe fatigue. Differences on demographic and medical characteristics between severely and non-severely fatigued cancer patients were tested with *χ*^2^. For the second objective, to find the contributing factors, two steps were taken. The first step was to test the differences between severely fatigued cancer patients and non-severely fatigued cancer patients on the contributing factors with a *t*-test for independent samples. For the second step a logistic regression analysis was performed using Stepwise Forward method. This method was chosen, as it was an exploratory data analysis. Significant factors found in the first step were put in the logistic regression as independent variables, with significant demographic and medical variables as covariates. The dimensions of depression and sleep were each measured with two instruments, although measuring different aspects. When both instruments showed significant results in the first step, the instrument with the largest significant difference was put into the logistic regression. Variables that applied to the period before diagnosis were entered into the first block, and variables that applied to the current period were entered into the second block. Two persons with missing data on the BDI-PC or SCL-90 were excluded from the analysis. A two-sided *P*<0.05 was considered significant.

## Results

**Figure 1:**
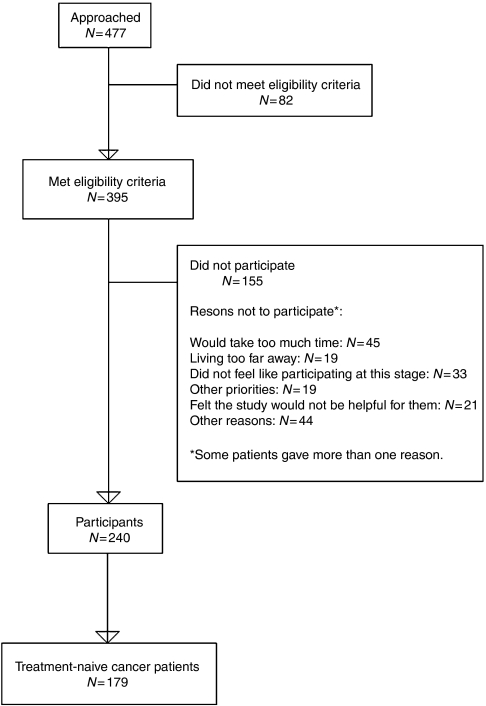
Flow chart showing the accrual of patients.

In total 477 patients agreed to be approached and were contacted by telephone. During the telephone conversations an additional 82 patients were excluded who did not meet the eligibility criteria. The most common reasons for exclusion were: having a co-morbidity that could cause fatigue, and being severely fatigued for several years. Of the 395 patients who met the inclusion criteria 155 refused to participate for different reasons (see Figure 1). In total 240 patients participated and completed baseline assessments. The characteristics of participants were compared with non-participants (see Table 1). Results showed that cancer patients who refused to participate were significantly older compared to patients who participated. No differences were found on gender or diagnosis between participants and non-participants.

Patients usually start with cancer treatment relatively fast after being diagnosed. As a consequence of this short time span baseline assessments sometimes took place when cancer treatment had just started. For example, some breast cancer patients were assessed after surgery, but before adjuvant radiotherapy or chemotherapy. Of the 240 participants 61 patients were assessed when their cancer treatments had just started, but their data were not used in the analysis. Thus data presented in this study are based on 179 cancer patients who were treatment naive.

### Patient characteristics

Most of the 179 patients were diagnosed with breast cancer or prostate cancer, 54% were female, and 82% were married (see Table 2). The mean age of the sample was 56.6 (s.d. 10.9) years, and the mean education level was 4.1 (s.d. 1.7) ranging between one and seven (data not shown). No differences were found between severely and non-severely fatigued cancer patients on demographic variables such as sex, age, education or marital status, although the difference between males and females nearly reached significance. In addition, differences were tested on several medical variables between severely fatigued cancer patients and non-severely fatigued cancer patients. No significant differences were found on current medication use, and on the medical history of co-morbidities, and receiving psychological or psychiatric treatment in patients’ lifetime (all *P*>0.971) (data not shown).

### The presence of severe fatigue in cancer patients before treatment

In the total sample 23.5% of the cancer patients were severely fatigued, but this percentage varied between diagnoses (see Table 2). The presence of severe fatigue was the lowest in patients with prostate cancer (14.3%), but higher in breast cancer patients (20.3%). In the group of patients with other tumours the presence of severe fatigue was the highest (33.3%). When patients with gastrointestinal cancer were considered as a separate group, fatigue in this specific group was 28.1%. In patients with other tumours without gastrointestinal cancer severe fatigue even rose to 38.2%. A significant overall effect of diagnosis on severe fatigue was found using the *χ*^2^ test (*P*=0.044). In addition, we tested if the means of the three diagnosis groups were different on the CIS using ANOVA, and also a significant overall effect was found (*P*=0.014). Using a *post hoc* test we tested which of the three groups (breast cancer (mean 23.5, s.d. 12.4), prostate cancer (mean 19.9, s.d. 12.0), or other tumours including gastrointestinal cancer (mean 27.1, s.d. 13.9)) differed from each other, and found one significant difference. Patients with prostate cancer were significantly less fatigued compared with the group of patients with other tumours (*P*=0.011) (data not shown).

### Contributing factors to severe fatigue before cancer treatment

In Table 3 the differences on contributing factors between severely fatigued cancer patients and non-severely fatigued cancer patients are described. Severely fatigued cancer patients reported to have more fatigue in the period before diagnosis, more pain, and being less physically active. These differences were significant for both periods, 1 and 3 years before diagnosis. Severely fatigued cancer patients also reported currently more pain and being less physically active, than non-severely fatigued cancer patients. In addition, they reported significantly more sleeping problems, and more feelings of depression and anxiety.

The results of the logistic regression are described in Table 4. Four factors contributed uniquely to severe fatigue in cancer patients before treatment. First fatigue 1 year before diagnosis contributed significantly. Three factors of the current period contributed significantly to severe fatigue. Lower physical activity contributed the most, followed by depressive mood and impairments on sleep and rest. Two factors, diagnosis and physical activity 3 years before diagnosis, did not contribute significantly to severe fatigue. Anxiety and pain were not part of the logistic regression.

## Discussion

This is the first study specifically aimed at investigating fatigue in patients who were recently diagnosed with cancer, before initiation of any treatment for cancer. The first goal of this study was to establish how many cancer patients report severe fatigue before receiving treatment. In the whole sample 24% of the cancer patients were severely fatigued, ranging from 14 to 28%. The presence of severe fatigue was the lowest in patients with prostate cancer (14%), but higher in breast cancer patients (20%), and gastrointestinal cancer patients (28%).

The prevalence of severe fatigue in our study is surprisingly high, in perspective to results in other samples. Reviewing the results of seven different studies fatigue in cancer survivors appear to vary between 16 and 38%, compared with 10–11% in a control group (Gielissen, 2007). The prevalence of severe fatigue in cancer survivors with various cancer diagnoses was about 22% (Servaes *et al*, 2001, 2002c; Bartsch *et al*, 2003). Thus severe fatigue in cancer patients before treatment seems two times as high compared with people without a history of cancer, and reaching the level of severe fatigue in cancer survivors long after cancer treatment.

As patients in this study were recruited as part of a larger intervention study, the question rises if this could be a biased sample. In the general Dutch population more males are diagnosed with cancer than females. In addition, breast cancer is the most common type of cancer, followed by colorectal cancer, lung cancer, and prostate cancer (Visser *et al*, 2003). So the sample in this study does not reflect the incidence and types of cancer in the Dutch population, as more females were included and prostate cancer was more common than gastrointestinal cancer in this sample. These differences cannot be explained from the characteristics of the patients who refused to participate, as no significant differences were found between participants and non-participants on sex and diagnosis. The following reasons might explain these differences. One reason might be, because this sample is a selected group of cancer patients who would be treated with curative intent. Another reason might be that patients with colorectal cancer are more often diagnosed in an acute phase requesting immediate treatment, whereas patients with breast or prostate cancer receive treatment in a more planned manner. Patients diagnosed and treated in this acute phase were more difficult to approach and to include into the study. This also explains the small numbers of patients with testis cancer in this study, who also receive surgery in an acute phase. The organisation of the recruitment might also explain the differences between our sample and the Dutch population. For example, in most hospitals specialised mamma care units were involved with recruiting patients, which might explain the large numbers of patients with breast cancer.

The question rose if severe fatigue was more common in this sample as patients were recruited for an intervention study on fatigue during cancer treatment. We do not expect that patients with severe fatigue are over-represented in this sample. Firstly, physicians excluded patients with co-morbidities that could have caused fatigue, for example patients with rheumatic arthritis or heart disease. Secondly, patients were informed that prevention of severe fatigue was the main goal of the study, and patients who indicated seeking help for severe fatigue were not included in the study. Thirdly, although patients were excluded when receiving psychiatric or psychological treatment in the last 3 months based on self-report, patients were not excluded based on taking psychotropic medicines. However, only one participant took psychotropic medicine, so it is improbable that this is an explanation for the prevalence of severe fatigue. The prevalence of severe fatigue might even be underestimated. More patients with prostate cancer (who are less frequently and severely fatigued), and less patients with colorectal cancer (who are more often severely fatigued) participated in this study, compared with the Dutch population.

Our second goal was to investigate which factors influenced severe fatigue before cancer treatment, and four factors were found. More fatigue 1 year before diagnosis, currently lower physical activity, depressive mood and more impaired sleep and rest appeared to be related to fatigue prior to treatment. Although differences were found in the prevalence of severe fatigue among various groups of diagnoses, results showed that diagnosis did not uniquely contribute to severe fatigue. In the light of this result, TNM classification of each tumour was considered not useful. Further classification would increase the number of subgroups, making it even harder to demonstrate a potential relationship between diagnosis and fatigue. The four mentioned factors are thus stronger related to severe fatigue than diagnosis, and also stronger than anxiety, pain or physical activity 3 years before diagnosis.

Lower physical activity was related to fatigue in cancer patients during treatment (Dimeo *et al*, 1997; Berger, 1998; Berger and Farr, 1999) and in cancer survivors (Bergner *et al*, 1981; Servaes *et al*, 2002c) as previous studies revealed. A new finding is that this relationship between physical activity and fatigue was now found in cancer patients before initiation of treatment.

Two studies that investigated the quality of life in newly diagnosed cancer patients found that sleeping problems affected patients before cancer treatment (Cimprich, 1999; Shepherd and Fisher, 2004); however, it remained unclear which aspects of sleep were affected. Looking at our results on the subscale sleep/rest of the SIP more closely revealed four differences between severely and non-severely fatigued cancer patients. Severely fatigued cancer patients indicated to sleep less at night, sleep or nap more during the day, sit during much of the day, and lie down to rest more often during the day. Thus, not only the nocturnal sleep was affected in severely fatigued cancer patients, but their daily sleep and rest was affected too.

One of the symptoms of clinical depression can be fatigue. However, the prevalence of clinical depression in our sample is low (6.2%) and within the normal range of the adult Dutch population (5.7–6.6%) (RIVM, 2007). Thus, clinical depression cannot be an explanation for the high prevalence of severe fatigue in this study. In addition our results showed the necessity to distinguish clinical depression from depressive mood, with the latter clearly being a mood state. Although no structured psychiatric interview was used to diagnose mood disorders.

Fatigue 1 year before diagnosis was evaluated retrospectively by patients, and not measured at that specific time. This evaluation probably reflects patients’ recollection of fatigue 1 year before diagnosis, rather than actual fatigue at that time.

Contrary to what was expected anxiety was not found as a fatigue-contributing factor. Receiving the diagnosis cancer can inflict strong feelings of anxiety (Stanton and Snider, 1993; Cimprich, 1999), although these high levels appeared to decrease within 2 weeks (Shepherd and Fisher, 2004). This trend was also found in patients receiving chemotherapy. High level of anxiety prior to chemotherapy decreased as soon as individuals started treatment (Jacobsen *et al*, 1993; Trask *et al*, 2003). Thus, feelings of anxiety are a common reaction on being diagnosed with cancer, and the prospect of receiving chemotherapy, but feeling of anxiety are not related to severe fatigue in treatment naive cancer patients.

No previous research was done to investigate the relationship between fatigue and pain in treatment naive cancer patients, but no evidence was found that pain contributed to severe fatigue in this study.

One of the limitations in this study is the reliance on cross-sectional data. Therefore, we cannot make any claims for causality between fatigue and the related factors, impaired sleep and rest, depressive mood, and physical activity.

In this study questionnaires were used to measure physical activity, but asking people to estimate their level of physical activity has its limitations. Previous research showed that there is a lack of correspondence between self-reported physical activity and objective physical activity (Vercoulen *et al*, 1997; Servaes *et al*, 2002b). Probably the perception of physical activity does not always reflect the actual level of physical activity. Thus in our study it is more likely that the perceived level of physical activity was related to severe fatigue rather than the actual level.

In summary, it is generally known that most cancer patients will experience fatigue during treatment, but fatigue in treatment naive cancer patients was not previously investigated. This study showed that a large number of cancer patients already experience severe fatigue before initiation of cancer treatment. One might expect that the course of fatigue during and after cancer treatment could be different for patients with severe fatigue or patients without severe fatigue before cancer treatment. In addition, it remains a question if patients with severe fatigue before cancer treatment should receive a kind of early fatigue intervention at this stage. These will be topics for future research.

## Figures and Tables

**Table 1:** Differences between participants and non-participants

	**Participants**	**Non-participants**	
**Characteristics**	**240**	**155**	
**Total (*n*)**	**Mean (s.d.)**	**Mean (s.d.)**	***P*-value**
Age (years)	56.8 (11.1)	59.7 (10.9)	**0.010**
			
*Sex*	*n* (%)	*n* (%)	0.219
Male	92 (38.3)	50 (32.3)	
Female	148 (61.7)	105 (67.7)	
			
*Diagnosis* a			0.310
Breast cancer	109 (45.4)	81 (52.3)	
Prostate cancer	57 (23.8)	28 (17.4)	
Other tumours	76 (31.7)	47 (30.3)	
Gastrointestinal	33	26	
Urogenital	16	9	
Gynaecological	13	10	
Lymphomas	7	0	
Sarcoma	3	2	
Melanoma	2	0	
Thyroid carcinoma	2	0	

a Three patients were diagnosed with both bladder and prostate cancer and were categorised as urogenital tumours of the other tumours. A two-sided *P*<0.05 was considered significant.

**Table 2:** Data of demographic variables, diagnosis and presence of severe fatigue

	**Total sample**	**Non-severely fatigued cancer patients**	**Severely fatigued cancer patients**	**Difference^a^**
**Characteristics**	***n***	***n* (%)**	***n* (%)**	***P*-value**
Total	179	137 (76.5)	42 (23.5)	
				
*Sex*				0.064
Male	82	68 (82.9)	14 (17.1)	
Female	97	69 (71.1)	28 (28.9)	
				
*Age (years)*				0.237
Younger age group (⩽57)	88	64 (72.7)	24 (27.3)	
Older age group (>58)	91	73 (80.2)	18 (19.8)	
				
*Education level*				0.269
Lower education (⩽4)	111	88 (79.3)	23 (20.7)	
Higher education (>4)	68	49 (72.1)	19 (27.9)	
				
*Marital status*				0.568
Married/cohabiting	146	113 (77.4)	33 (22.6)	
Other status (unmarried/divorced/widowed)	33	24 (72.7)	9 (27.3)	
				
*Diagnosis* b				**0.044**
Breast cancer	64	51 (79.7)	13 (20.3)	
Prostate cancer	49	42 (85.7)	7 (14.3)	
Other tumours	68	46 (67.7)	22 (33.3)	
Gastrointestinal	32	23 (71.9)	9 (28.1)	
Urogenital	14		6	
Gynaecological	10		3	
Lymphomas	5		2	
Sarcoma	3		1	
Melanoma	2		1	
Thyroid carcinoma	2		1	

a Difference between severely and non-severely cancer patients, tested with χ^2^.

b Two patients were diagnosed with both bladder and prostate cancer and were categorized as urogenital tumours of the other tumours. A two-sided *P*<0.05 was considered significant.

**Table 3:** Contributing factors to severe fatigue before cancer treatment tested with a *t*-test

**Factors**	**Non-severely fatigued mean (s.d.)**	**Severely fatigued mean (s.d.)**	***t***	**d.f.**	***P*-value**
*Period before diagnosis*					
Fatigue 1 year before diagnosis^a^	1.71 (2.43)	3.86 (2.89)	4.786	177	<0.001
Fatigue 3 years before diagnosis	1.32 (2.14)	2.93 (2.67)	3.563	177	0.001
Physical activity 1 year before diagnosis	7.06 (2.34)	6.17 (2.66)	−2.094	177	0.038
Physical activity 3 years before diagnosis^a^	7.28 (2.24)	6.17 (2.63)	−2.711	177	0.007
Pain 1 year before diagnosis	0.89 (1.91)	1.95 (2.68)	2.391	177	0.020
Pain 3 years before diagnosis^a^	0.73 (1.66)	1.76 (2.43)	2.577	177	0.013
					
*Current period before cancer treatment*					
Pain^a^	1.28 (2.14)	3.24 (3.04)	3.900	177	<0.001
Anxiety^a^	13.1 (3.77)	17.0 (6.91)	3.502	176	0.001
Sleep quality (SCL-sleep)	4.91 (2.23)	6.91 (2.93)	4.061	176	<0.001
Impairments on sleep/rest (SIP-SR)^a^	34.7 (46.4)	85.1 (70.4)	4.358	177	<0.001
Physical activity^a^	6.66 (2.42)	4.69 (2.37)	−4.636	177	<0.001
Depressive mood (SCL-90)^a^	20.8 (5.86)	27.5 (9.54)	4.360	176	<0.001
	% (*n*)	% (*n*)			
Clinical depression (BDI-PC)^b^	2.9 (4)	17.1 (7)			0.004

a Factors that were put into the logistic regression analysis as separate factors.

b Difference on clinical depression was tested with Fisher's Exact Test.

**Table 4:** Contributing factors to severe fatigue

**Contributing factors**	**B (SE)**	**Exp b**	***P*-value**	**95% CI**
Diagnosis group			0.374	
Diagnosis group (1) (breast cancer)	0.783 (0.609)	2.187	0.199	0.663–7.218
Diagnosis group (2) (prostate cancer)	0.270 (0.606)	1.310	0.656	0.399–4.297
				
*Period before diagnosis*				
Fatigue 1 year before diagnosis	0.218 (0.077)	1.244	**0.005**	1.070–1.446
Physical activity 3 years before diagnosis	0.020 (0.111)	1.020	0.855	0.822–1.267
				
*Current period before treatment*				
Depressive mood (SCL-90)	0.076 (0.031)	1.079	**0.014**	1.015–1.147
Impairments on sleep/rest (SIP-SR)	0.008 (0.004)	1.008	**0.045**	1.000–1.015
Physical activity	−0.284 (0.115)	0.752	**0.013**	0.601–0.943
Constant	−2.986 (1.040)	0.051	0.004	

(*R*^2^ was 0.274 (Cox & Snell) and 0.412 (Nagelkerke)). A two-sided *P*<0.05 was considered significant.
